# Common Variable Immunodeficiency Patient Fecal Microbiota Transplant Recapitulates Gut Dysbiosis

**DOI:** 10.21203/rs.3.rs-2640584/v1

**Published:** 2023-03-13

**Authors:** Joud Hajjar, Anita Voigt, Margaret Conner, Alton Swennes, Stephanie Fowler, Chadi Calarge, Danielle Mendonca, Dominique Armstrong, Cheng-Yen Chang, Jolan Walter, Manish Butte, Tor Savidge, Julia Oh, Farrah Kheradmand, Joseph Petrosino

**Affiliations:** Baylor College of Medicine

**Keywords:** Common Variable Immunodeficiency, Microbiome, Fecal Microbiome Transplant, Germ Free Mice

## Abstract

**Purpose:**

Patients with non-infectious complications have worse clinical outcomes in common variable immunodeficiency (CVID) than those with infections-only. Non-infectious complications are associated with gut microbiome aberrations, but there are no reductionist animal models that emulate CVID. Our aim in this study was to uncover potential microbiome roles in the development of non-infectious complications in CVID.

**Methods:**

We examined fecal whole genome shotgun sequencing from patients CVID, and non-infectious complications, infections-only, and their household controls. We also performed Fecal Microbiota transplant from CVID patients to Germ-Free Mice.

**Results:**

We found potentially pathogenic microbes *Streptococcus parasanguinis* and *Erysipelatoclostridium ramosum* were enriched in gut microbiomes of CVID patients with non-infectious complications. In contrast, *Fusicatenibacter saccharivorans* and *Anaerostipes hadrus*, known to suppress inflammation and promote healthy metabolism, were enriched in gut microbiomes of infections-only CVID patients. Fecal microbiota transplant from non-infectious complications, infections-only, and their household controls into germ-free mice revealed gut dysbiosis patterns in recipients from CVID patients with non-infectious complications, but not infections-only CVID, or household controls recipients.

**Conclusion:**

Our findings provide a proof of concept that fecal microbiota transplant from CVID patients with non-infectious complications to Germ-Free mice recapitulates microbiome alterations observed in the donors.

## Introduction

Common variable immunodeficiency (CVID) is the most common treatable inborn error of immunity in adults (1, 2). It is characterized by low immunoglobulin (Ig) levels (IgG, IgA, and/or IgM) and recurrent infections due to B-cell defects (3). Clinically, CVID patients present with two broad phenotypes: those with infections-only (INF) and those with additional autoimmune and autoinflammatory complications known as non-infectious complications (NIC) (1, 4, 5). Nearly 60% of CVID patients develop NIC, which manifests as cytopenia, inflammatory bowel disease (IBD)-like disease, chronic lung disease, and lymphoproliferation (1, 6-9). In addition, NIC-CVID patients have a significant increase in morbidity and mortality compared to INF-CVID patients (10-12). Thus, there is a pressing need to improve our understanding of NIC-CVID.

Several recent studies have suggested involvement of the gut microbiome in CVID-associated immune dysregulation. Specifically, bacteria and their associated products translocate across “leaky” gut epithelium into systemic circulation, as evidenced by the detection of circulating lipopolysaccharide (LPS) or bacterial DNA(13-15). Furthermore, LPS activates an immune response through the recognition of microbe-associated molecular patterns (16, 17), releasing pro-inflammatory cytokines (18). Other clinical studies have also shown that the gut microbial composition is altered in CVID patients (i.e., dysbiosis), particularly in NIC-CVID (14, 19, 20). Furthermore, 16S ribosomal RNA (rRNA) gene sequencing data from CVID patient stool samples showed lower within-sample taxonomic diversity (i.e., alpha diversity) compared to controls (19, 21). Reduced alpha diversity and increased circulating LPS concentration are also more common in NIC-CVID compared to INF-CVID patients, suggesting that the translocation of certain bacteria may be implicated in immune dysregulation observed in NIC-CVID (22). In fact, IgG replacement reduces circulating LPS concentrations, suggesting it may reduce gut bacterial translocation (13), or that polyclonal IgG blocks LPS activity in other ways. Additionally, when mucosal integrity is disrupted, some pathobionts, such as *Acinetobacter baumanni*, induce inflammation by triggering mucosal intestinal macrophages to produce inflammatory cytokines (23). However, it remains unclear if the gut microbiome in NIC-CVID patients is distinct from that in INF-CVID patients and whether NIC-CVID gut dysbiosis can be recapitulated in animal models. Additionally, microbiome diversity, enrichment, and the specific taxa linked to CVID phenotypes remain unclear.

To address these questions, we examined the gut microbiome composition in NIC-CVID and IFN-CVID patients, as well as their household controls. First, we established the baseline composition of the INF-CVID and NIC-CVID microbiomes at species-level resolution. Then, we comprehensively assessed the gut microbiome using metagenomic whole genome shotgun sequencing (mWGS) from 11 CVID patients (6 NIC-CVID and 5 INF-CVID) and their household controls. Finally, because CVID is a rare disease with no widely accepted animal models, we performed fecal microbiota transplant (FMT) from these CVID patients and household controls into germ-free C57Bl/6J GF mice to assess the feasibility of modeling CVID gut dysbiosis in mice.

## Methods

### Recruitment Of Cvid Patients

Patients were diagnosed with CVID by their treating clinical immunologists. Table S1 summarizes patients' characteristics. We excluded patients on immune suppressive medications, with an acute infection/illness, and those who received antibiotics 30 days before enrollment. We defined NIC-CVID patients as having severe forms of autoimmunity/immune dysregulation associated with CVID (1) (i.e., Granulomatous interstitial lung disease, colitis, nodular regenerative hyperplasia, lymphoproliferation, and severe cytopenia). Common autoimmunity, such as hypothyroidism alone, was not considered NIC.

### Fecal Microbiome Transplant

FMT experiments were performed as described previously (24). Fecal matter was thawed, diluted (100 mg/1 ml sterile PBS), passed through a 40μm strainer thrice, and then frozen at − 80°C. GF*(C57BL/6J)* mice (males and females, age 8–12 weeks) were orally gavaged (2–3 times over 1 week at 200μl/dose) with fecal matter from either NIC-CVID or INF-CVID patients or a healthy donor. Mice were allowed 30 days for the microbiome to stabilize. Blood and feces were collected at baseline and 30 days later.

### Whole Genome Shotgun Sequencing

Libraries were prepared using the Nextera XT DNA Library Prep Kit (Illumina, San Diego, CA, USA) according to the manufacturer's instructions, except for using one-quarter of the recommended reaction volume. WGS was predominantly carried out using a 2× 150bp (paired-end) sequencing protocol on the NovaSeq 6000 Sequencing System (Illumina), according to the manufacturer's manual. Sequencing was conducted at the Genome Technologies core facility at the Jackson Laboratory for Genomic Medicine, Farmington, USA.

### Data Processing

Samples with fewer than 1.4 million reads were excluded, leaving 22 samples from CVID patients, 15 household controls, and 112 mouse fecal samples for analysis. Relative proportions were used for all analyses. All taxonomic features at the species level with a mean relative abundance of 0.01% (denoise function, (25) across the dataset were removed from the dataset to reduce potential false positives and allow for multiple hypothesis correction.

### Biomarker Discovery With Lefse

LEfSe pipeline (26) was used with default parameters (LDA score log(10) > 2.0) to identify discriminant taxa between sample groups. We opted to categorize our metagenomic profiles based on MetaPhlAn 4. MetaPhlAn 4 is a tool for profiling microbiome communities and uses a database of unique, clade-specific gene markers. It assigns fragments by mapping them against the gene markers database (27). MetaPhlAn is associated with higher accuracy and lower rates of false positivity (27, 28).

### Statistical analysis

We used the R-based software Agile Toolkit for Incisive Microbial Analyses (ATIMA) (29) to generate plots visualizing alpha diversity (richness and evenness), beta diversity (in-between sample differences), and taxa abundances (phylum-genus) through box plots, Principal Coordinate Analysis (PCoA) ordinations, and heatmaps. ATIMA enables rarefied and non-rarefied relative abundance analysis. We analyzed categorical variables using the non-parametric Mann-Whitney and Kruskal-Wallis tests for variables with 2 groups or ≥ 3 groups, respectively. P-values were adjusted for multiple comparisons using the FDR (false discovery rate) algorithm.

The inter-group dissimilarities (beta diversity) in gut microbiota composition were measured using the Bray–Curtis distance metrics. Bray Curtis dissimilarity quantifies the differences in species populations between two different sites. The resulting number is between 0 and 1, with 0 denoting the highest similarity (two samples share the same species) and 1 denoting the highest dissimilarity.

## Results

### Gut microbiome alpha diversity is comparable between NIC-CVID and INF-CVID patients, as well as their household controls

**Figure 1:**
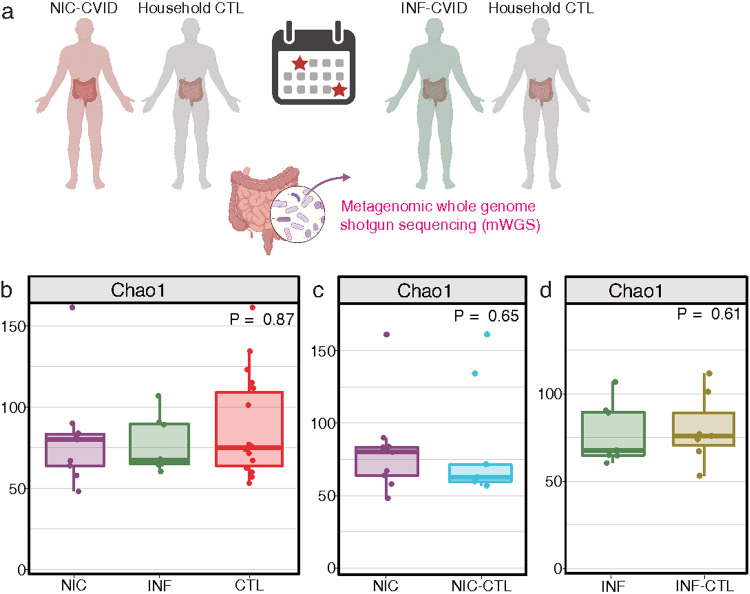
Richness of the gut microbiome did not significantly differ between CVID patients and their household control. **(a)** Schematic of the study design for sample collections from CVID patients and household control (CTL). A total of 11 patients with CVID (6 NIC-CVID, 5 INF-CVID), along with their household controls were included. Each patient and control provided 2 samples, 20 days apart. Stool samples were collected at home, freshly frozen, and then shipped on ice. **(b)** Microbial alpha diversity is not statistically significantly different between NIC-CVID patients, INF-CVID patients, and household controls. **(c)** No significant differences between NIC-CVID and their matched household CTL (NIC-CTL) or **(d)** INF-CVID with their household controls (INF-CTL). The analysis was performed by Kruskal–Wallis H test in **b** and by the Mann-Whitney U test in **c** and **d**. Figure 1a was created with BioRender.com

Greater diversity within each sample, known as alpha diversity, is often associated with a stable microbiome and healthy metabolism (30, 31). To determine the effect of CVID on gut microbiome richness and diversity, we performed mWGS on the gut microbiome of NIC-CVID and INF-CVID patients, as well as their household controls (Fig. 1a). We found microbial alpha diversity was not statistically significantly different between NIC-CVID and INF-CVID patients or their household controls. Still, notably, Alpha diversity in the NIC-CVID participants was qualitatively lower compared to INF-CVID, and household controls (Fig. 1b). Nor did we detect any significant differences in alpha diversity between NIC-CVID and their matched household control or between INF-CVID and their matched household control were observed (Figs. 1c and d).

### Nic-cvid And Inf-cvid Patients Exhibit Dissimilar Gut Microbiome Composition

**Figure 2:**
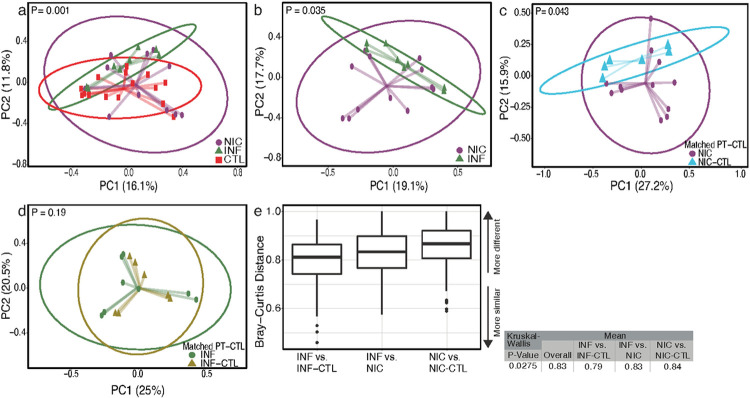
NIC- and INF-CVID exhibit dissimilar gut microbiome compositions. Beta diversity between **(a)** NIC-CVID, INF-CVID, and household CTL, **(b)** NIC- and INF-CVID, **(c)** NIC-CVID and their own household CTL, **(d)** INF-CVID and their own household CTL. **(e)** Inter-group dissimilarities in gut microbiota composition between CVID patients and household CTL showed that the NIC-CVID group has a greater variation in gut microbiota than other groups (P=0.027). Principal-coordinate analysis plots of Bray-Curtis distances of fecal microbiota generated from mWGS sequence analysis. PC1, principal coordinate 1; PC2, principal coordinate 2. Analysis used weighted Bray-Curtis in **a** and **b,** unweighted Bray-Curtis in **c** and **d,** and the Kruskal-Wallis test in **e.** In e, only the statistically significant dissimilarities were included. Closer to 1 means the samples are more dissimilar. Total of 6 NIC, 4 NIC-CTL, 5 INF, 4 INF-CTL.

Beta diversity captures differences in microbiota composition between two groups (32). To identify potential associations between gut microbial composition and CVID phenotype, we used the Bray–Curtis dissimilarity matrix to cluster the metagenome using ATIMA (Agile Toolkit for Incisive Microbial Analysis), developed by the Center for Metagenomics and Microbiome Research at Baylor College of Medicine (29, 33). CVID patients' bacterial microbiomes clustered separately from household controls and INF-CVID patients (Figs. 2a and 2b).

Next, we compared each CVID phenotype with their household controls. The microbial composition of NIC-CVID patients was distinct from that of their household controls (Fig. 2c), whereas the microbiota composition of INF-CVID patients did not significantly differ from that of their household controls (Fig. 2d).

We next compared inter-group dissimilarities in gut microbiota composition. We found the NIC-CVID group had greater microbiota variation from their household controls compared to the other groups (Fig. 2e). These findings indicate that NIC-CVID is associated with a significant shift in gut microbiome composition that overcomes the similarities that can be shared due to kinship and diet (34-36).

### Distinct Microbial Species Are Associated With Nic-cvid And Inf-cvid Patients

**Figure 3:**
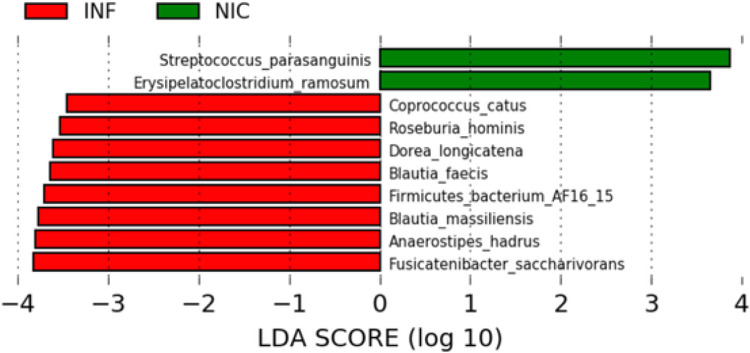
Linear discriminant analysis (LDA) of differentially abundant species in fecal microbiota between NIC- and INF-CVID. This analysis revealed significant differences in the gut microbiome of NIC-CVID compared to INF-CVID. LDA scores (log10) > 2 and p-value < 0.05 are shown.

We used linear discriminant analysis (LDA) and LDA effect size (LEfSe) to identify microbes differentially associated with NIC-CVID or INF-CVID (26). LEfSe couples standard tests for statistical significance with additional tests encoding biological consistency and effect relevance to determine the features, such as organisms, clades, operational taxonomic units, genes, or functions, most likely to explain differences between classes (26). We found significant differences in the gut microbiome composition of NIC-CVID and INF-CVID patients at the species level (Fig. 3). The discriminant species for the NIC-CVID group were *Streptococcus parasanguinis* and *Erysipelatoclostridium ramosum*. Both are pathobionts reported to cause severe infections in immunocompromised hosts (37, 38). In contrast, the microbiome of INF-CVID patients showed a preponderance of several microbes associated with anti-inflammatory effects, including *Fusicatenibacter saccharivorans, Dorea longicatena*, and *Blautia faecie* (39-41). Additionally, we identified in the gut microbiome of INF-CVID patients an enrichment of microbes that are associated with healthy metabolism, including *Anaerostipes hadrus* (42), *Coprococcus catus* (43), and *Roseburia hominis* (44).

### A New Cvid-fmt Gut Dysbiosis Model In Gf Mice

**Figure 4:**
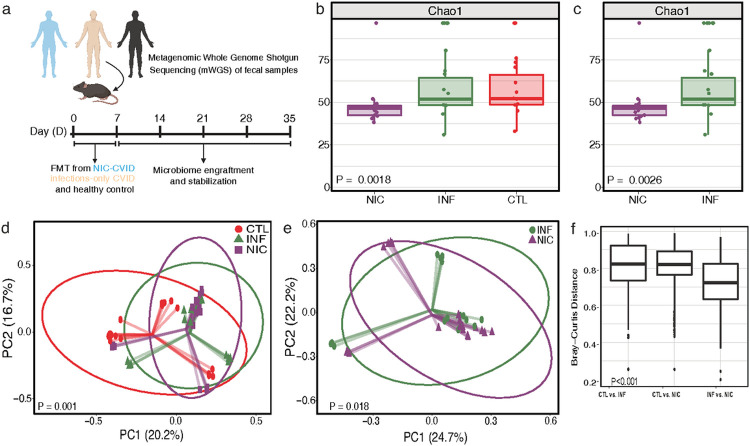
FMT from CVID patients to GF mice recapitulates CVID patients' gut dysbiosis. **(a)** Experimental design of FMT from NIC- and INF-CVID patients and household control (CTL) to Germ-Free (GF) (C57BL/6J) mice. Mice were orally gavaged (2-3 times for 1 week at 200μl/dose) with fecal matter. Mice were allowed 30 days for the microbiome to stabilize. **(b)** Alpha diversity between NIC, INF, and CTL FMT recipients. **(c)** Alpha diversity between NIC- and INF-FMT recipients. Beta diversity between NIC-FMT, INF-FMT, and CTL-FMT. **(d)** Beta diversity between NIC-FMT, INF-FMT, and CTL-FMT recipients. **(e)** Beta Diversity between NIC-FMT and INF-FMT recipients only. Axis labels indicate the percentage of variance explained by respective principal coordinate axis. PC1, principal coordinate 1; PC2, principal coordinate 2. Donors (NIC-CVID n=3, INF-CVID n=3, and CTL n=3). GF mice aged 8-12 weeks. NIC-FMT n= 19 (16 males, 3 females), INF-FMT n=16 (12 males, 4 females), CTL-FMT n=17 (13 males, 4 females). The analysis was performed by Kruskal–Wallis H test in **b** and **c** and by the Mann-Whitney U test in **d** and **e**. **(f)** Inter-group dissimilarities in gut microbiome composition between FMT recipients were most notable between FMT recipients from patients with CVID and their household controls (CTL) FMT recipients, the closer to 1 means the samples are more dissimilar. Analysis was performed using the Kruskal-Wallis test. Figure 4a was created using BioRender.com

Although CVID is considered the most common treatable inborn error of immunity in adults, it is still a rare and heterogeneous disease. A broader understanding of the role of the gut microbiome and its impact on immune regulation, in CVID patients, remains unclear. To determine the degree to which FMT would recapitulate differences in microbial composition observed in our human participants, we compared microbial communities between fecal matter from CVID patients, household controls, and FMT-recipient mice (Fig. 4a).

GF mice have low serum and fecal IgA and underdeveloped Peyer patches, as well as small and underdeveloped mesenteric lymph nodes (45). In addition, introducing normal flora into GF mice restores their capacity to produce mucosal and systemic immune responses (46). Consistent with these findings, our pilot studies showed that GF(C57Bl/6J) mice had undetectable serum IgA, variable serum IgG, and low fecal IgA/IgG levels (0–10 μg/ml and 0–3 ng/ml, respectively) at baseline (Figure S1a, b). Four weeks following FMT, serum IgA levels increased in all FMT recipients (Figure S1c). In addition, serum IgG increased, (Figure S1d), whereas fecal IgG levels remained low (0–6 ng/ml, similar to fecal IgG levels in WT C57Bl/6J mice) housed in a specific pathogen-free facility. We noted interesting differences when we compared the immunoglobulin levels between FMT groups. First, there was no significant difference in serum IgA among FMT recipients following FMT (Figure S1e). In contrast, total serum IgG was higher in both NIC-FMT and INF-FMT recipients, compared to CTL-FMT recipients. (Figure S1f). Notably, the increase in IgG subclasses differed per FMT group. IgG2b was significantly higher in both NIC-FMT and INF-FMT recipients compared to CTL-FMT (Figure S1g), while IgG2c was higher in INF-CTL compared to all other groups (Figure S1h). We measured IgG2c instead of IgG2a because C57BL/6 mice produce this isotype in place of IgG2a (47).

IgG2c in mice is produced as a result of Th1 response and INFγ production (48, 49), while IgG2b binds to FcγRIII and IV, activating FcγRs, which has been shown to induce autoimmunity, such as arthritis (50) and thrombocytopenia (51). Although, taken together, the antibody responses in CVID-FMT recipients may indicate an inflammatory response to FMT compared to CTL-FMT recipients, the findings should be interpreted with caution and require replication.

To prevent the development of anti-commensal antibody responses in FMT recipients, (52), we pretreated GF mice with 100 μg anti-mouse CD20 mAb intraperitoneally every two weeks, to prevent the development of anti-commensal antibody responses in FMT recipients, (52). Figure S2a and b show our flow cytometry gating strategy to assess mouse blood for B-cells before and after anti-CD20 depletion. Figure S2c shows successful B-cell depletion following anti-CD20 treatment. With this approach, we induced relative hypogammaglobulinemia (Figure S3a-d). The rationale for B-cell depletion is to prevent the production of specific antibodies to new antigens (53), generating a humoral immune defect that resembles CVID. No significant differences in FMT engraftment or mouse health were noted in mice treated with anti-CD20 mAb.

### Fmt From Cvid Patients To Gf Mice Recapitulates Cvid Patients' Gut Dysbiosis

We examined broad community metrics, including alpha and beta diversity, to characterize the overall similarity between donor and recipient communities. Four weeks following FMT, there was a significant difference in microbial richness and alpha diversity between NIC-FMT, INF-FMT, and CTL-FMT recipients (Fig. 4b). We also found a significant difference in gut microbial richness and alpha diversity between NIC-FMT and INF-FMT recipients (Fig. 4c). In addition, beta diversity measurements using unweighted and weighted UniFrac distances revealed that the gut microbiome composition was significantly different between the three FMT groups (Fig. 4d). Notably, the microbiota composition of NIC-FMT recipients was distinct from INF-FMT recipients (Fig. 4e). In addition, inter-group analysis in gut microbiota composition identified dissimilarities between FMT recipients, most notably between CVID-FMT and control-FMT recipients (Fig. 4f). Taken together, these results demonstrate that GF-FMT mouse recipients predominantly exhibited gut microbiome compositional aberrations resembling what was seen in CVID donors.

**Figure 5:**
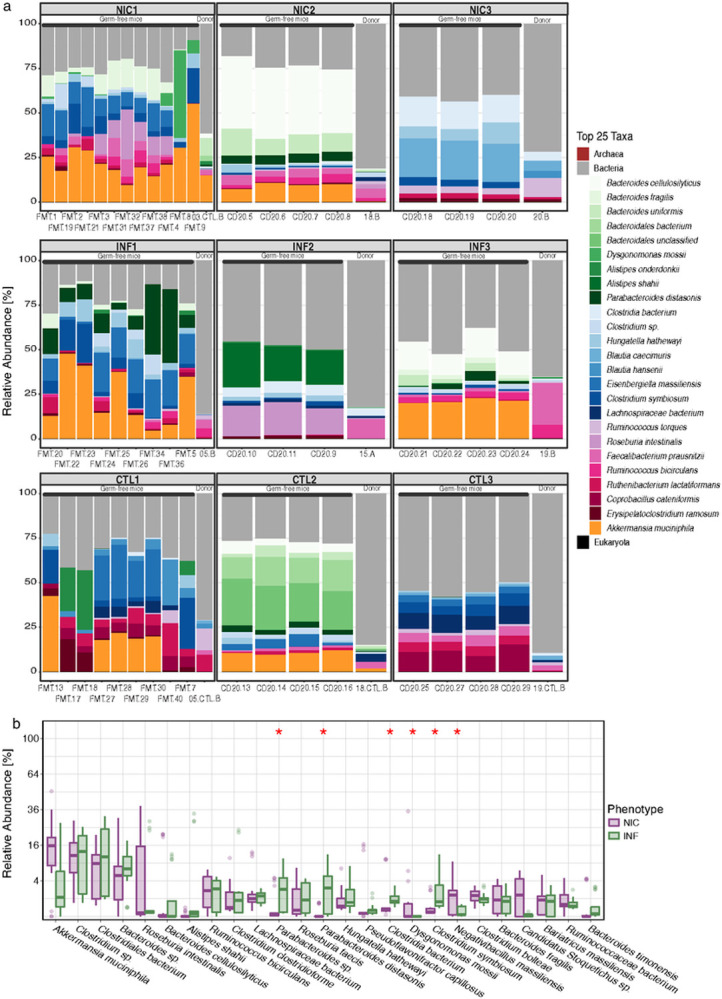
Relative abundance plots comparing human microbiome from donors to FMT-recipient mice (Top 25 taxa). **(a)** Comparison between each human donor and mouse 4 weeks following FMT indicates no statistically significant difference in the relative abundance between human donors and the mouse FMT recipient. Each FMT is represented here, including the human donor (NIC, INF, or CTL) and the recipient FMT group. The graph above is a representation of the top 25 taxa. (**b)** Comparison between NIC-FMT and INF-FMT microbes' relative abundance 4 weeks following FMT. The graph is representative of the top 25 taxa. Significant p-value <0.05 (marked by an asterisk).

We compared the relative abundance of the top 25 most abundant taxa between human fecal donors and FMT recipients (Fig. 5a). There was no statistically significant difference in the relative abundance between human donors and their respective FMT recipient mice in any of the FMT experiments. An exception was *Klebsiella sp.*, which was present in low abundance in one NIC-CVID patient but was not detected in the mice. *Bacteroides sp, Clostridium sp.*, and *Akkermansia muciniphila* had the highest relative abundance in both humans and mice.

Finally, we examined species-level differences between NIC-FMT and INF-FMT recipients. A representation of the mice's fecal microbiome that compares the relative abundance of the top 25 most abundant taxa between NIC-FMT and INF-FMT recipients is shown in (Fig. 5b). Similar to what we observed in CVID patients, NIC-FMT recipients had a higher relative abundance of microbes that can potentially cause opportunistic infections in immunocompromised individuals, including *Dysgonomonas mossii* and *Negativebacillus massiliensis. D. mossii* is a Gram-negative, anaerobic, coccobacillus-shaped bacteria within the phylum Bacteroidetes that has been reported to cause opportunistic infections in patients with type 1 diabetes and cancer (54-56). Similarly, *N. massiliensis* is a rare microbe that caused meningitis in a patient with Whipple Syndrome (57). On the other hand, INF-FMT recipients had a higher relative abundance of potentially beneficial microbes, including *Clostridium symbiosum* and *Parabacteroides distasonis. C. symbiosum* is a short-chain fatty acid producer associated with immunomodulatory and anti-inflammatory effects (58). Adding *C. Symbiosum* to the microbiota of a malnutrition mouse model ameliorated growth and metabolic abnormalities in the recipient mice (59). *P. distasonis* is one of 18 core members in the human gut microbiota (60) and thought to have critical physiological functions in its hosts. *P. distasonis* produces succinate (which activates gut glucogenesis) and transforms primary bile acids into secondary bile acids (61). Both succinate and secondary bile acids can promote gut barrier integrity and reduce inflammation in the gut of obese mice (62).

Taken together, our mWGS analysis of fecal matter from CVID patients and FMT-recipient GF mice revealed a high level of similarity between humans and mice, both in diversity metrics and in potential function. Both NIC-CVID patients and NIC-FMT recipients harbored potential pathogenic microbes associated with opportunistic infections in immunocompromised hosts, whereas INF-CVID patients and INF-FMT recipients harbored microbes with beneficial metabolic functions and potential anti-inflammatory capacity.

## Discussion

In the present study, we performed mWGS on the gut microbiomes from NIC-CVID and INF-CVID patients, as well as their healthy household controls. To overcome intra-individual microbial variations that can be missed when only a single sample collection is used, we collected two samples from each patient and household control for a more accurate assessment of the microbiome composition (63). Additionally, we included healthy household members as a control for diet and environmental factors (64). Flousehold members share more of their gut microbes compared to unrelated individuals, and intimate partners share even more gut microbiota than other household members (35, 65). Using these robust methods, we were able to further characterize gut microbiome composition in CVID patients. We identified specific microbes that were more abundant in NIC-CVID patients, including *S. parasanguinis* and *E. ramosum. S. parasanguinis* is predominantly an oral cavity microbe that belongs to the viridans group streptococci (VGS). Although VGS are generally considered to be of low pathogenic potential in immunocompetent individuals, they can cause invasive diseases such as endocarditis, intra-abdominal infection, and shock (66). *S. parasanguinis* is known to produce hydrogen peroxide (67) and has been reported to cause invasive infections, such as infective endocarditis and pneumonia, in immunocompromised hosts (37, 68). Additionally, the presence of *S. parasanguinis* in the gut is associated with dysbiosis in inflammatory bowel disease patients, owing to oxidative stress resistance in such bacteria (69). Hence, it is plausible that *S. parasanguinis* contributes to gut dysbiosis and immune dysregulation in NIC-CVID. We also found that *E. ramosum* is more abundant in the gut microbiome of NIC-CVID patients. *E. ramosum* belongs to the Clostridia group and has been shown to cause severe infections, particularly in immunocompromised patients (38). Interestingly, *E. ramosum* produces an IgA protease that is capable of cleaving human IgA (70). *E. ramosum* has been shown recently to be over 80-fold enriched in individuals with selective IgA deficiency, especially in those with autoreactive anti-IgA antibodies, suggesting a potential role for this pathobiont as an autoimmune trigger (71).

In INF-CVID patients, we noted an increased abundance of several microbes associated with potential anti-inflammatory effects, including *F. saccharivorans, D. longicatena*, and *B. faecie*. We also identified microbes associated with healthy metabolism, including *A. hadrus, C. catus, R. hominis, Blautia massiliensis*, and *Firmicutes bacterium*.

The most abundant bacteria in INF-CVID patients was *F. saccharivorans*, a species of the *Clostridia* class. Its abundance is associated with ulcerative colitis remission (39). In contrast, its decrease is associated with increased ulcerative colitis disease activity, which has been attributed to its immunomodulatory effects and its ability to induce IL-10 production in humans and mice (39, 72). Similarly, the presence of *D. longicatena* in the gut microbiome is associated with Crohn's disease remission (40). The second most abundant bacteria in the gut of INF-CVID patients was *A. hadrus*, a human-derived butyrate-producing strain. In contrast, *A. hardus* was shown in mice to be beneficial by increasing butyrate levels in the gut and harmful by potentially causing worse chemically-induced colitis (42). Butyrate is produced when gut microbes ferment dietary fiber and is considered a health-promoting molecule due to its anti-inflammatory (73) and anti-neoplastic potential (74). We also revealed that two of the *Blautia* species were enriched in the INF-CVID gut microbiome. *Blautia sp* can metabolize polymethoxyflavones, which are major bioactive flavonoids with various biological activities, including anti-inflammatory and anti-cancer effects (41, 75). Finally, we observed *Firmicutes* was enriched in INF-CVID patients. Two studies that used 16S rRNA gene sequencing for CVID gut microbiomes identified an increase in some *Firmicutes* in CVID (14, 76) metabolize polymethoxyflavones, which are major bioactive flavonoids with various biological activities, including anti-inflammatory and anti-cancer effects produces butyrate and supports healthy metabolism (77). Notably, *Firmicutes* harbors H_2_-oxidizing properties that promote more efficient energy extraction from food (78). Although an abundance of *Firmicutes* in the gut microbiome is associated with obesity (77, 79), this property of *Firmicutes* might be beneficial in CVID patients, as many with enteropathy develop malnutrition (80). Overall, the gut microbiome in INF-CVID patients was enriched with microbes that have been associated with a healthy metabolism and anti-inflammatory effects. In contrast, the NIC-CVID microbiome was enriched with inflammation-associated microbes, especially in the immunocompromised host.

In addition to our comprehensive characterization of the CVID gut microbiome, we provided a proof of concept that FMT from CVID patients to GF mice recapitulates the microbiome alterations seen in CVID patients. As far as we are aware, our model is the first to use B-cell depletion, to induce hypogammaglobulinemia, and prevent the generation of specific antibody response against transplanted human microbiota, creating an antibody defect that resembles CVID immunophenotype. Even though the highest abundance of microbes in mice was not the same as in CVID patients, we noted the same potential pathogenicity and function in the gut microbiome of both mice and humans. The relative abundance of microbes associated with opportunistic infections and potential pro-inflammatory capacities were enriched in the NIC-CVID patients. On the other hand, microbes associated with a healthy metabolism and potential anti-inflammatory capacities were enriched in INF-CVID patients and INF-FMT recipients. In future studies, we believe this model may allow us to assess the impact of microbiome manipulation on immune responses and test therapeutics to ameliorate microbiome-associated immune dysregulation in CVID patients.

Although we did not detect a significant difference in alpha diversity between CVID patients and household controls, or between NIC-CVID and INF-CVID, we noted that Alpha diversity in the NIC-CVID participants was qualitatively lower compared to INF-CVID, and household controls. Previous studies showed that alpha diversity was lower in CVID patients compared to a general population healthy control and household controls using 16S rRNA gene sequencing (14, 20). However, smaller studies using mWGS showed that CVID patients (with no significant complications) had increased bacterial diversity compared to their household controls (81). Unlike 16S rRNA sequencing, mWGS reads all genomic DNA in a sample, rather than just one specific region of DNA, which allows the identification and profiling of all microbial genes present in the sample (the metagenome). Thus, metagenomic profiling often provides species-level assignment (82).

Our study has some limitations. Owing to the rare nature of inborn errors of immunity, this study comprised a small sample size. Also, our strict exclusion criteria eliminated patients with acute illness, infection, or recent use of antimicrobial agents. However, our longitudinal design mitigated these limitations to a degree. Additionally, household controls allowed us to control for shared diets and environments (31, 35). Finally, assessing two samples from each subject helps overcome some intra-individual microbial variations and provides a more accurate assessment of the microbiome composition (63).

Our goal for this study was not to generate a CVID mouse model but rather to create a gut dysbiosis model that could potentially be used to further model mucosal immune dysregulation in CVID. In addition, the model developed in this study may allow us to assess the impact of microbiome manipulation on immune responses and test therapeutics to ameliorate immune dysregulation in an immunocompromised host.

In conclusion, we demonstrated shifts in the gut microbiome of CVID patients. Specifically, we revealed that the microbiota in INF-CVID are potentially less pathogenic and have higher anti-inflammatory capacity compared to NIC-CVID.

## Data Availability

The datasets generated during and/or analyzed during the current study are available from the corresponding author on reasonable request.

## Abbreviations

16S rRNA: 16S ribosomal RNA
ATIMA: Agile Toolkit for Incisive Microbial Analysis
CTL: Control
CVID: Common Variable Immunodeficiency
FMT: Fecal microbiota transplant
GF: Germ Free
IBD: inflammatory bowel disease
INF: Infections-only
LEfSe: Linear discriminant analysis effect size
LDA: Linear discriminant analysis (LDA)
LPS: Lipopolysaccharide
mWGS: metagenomic whole genome shotgun sequencing
NIC: Non-infectious complications
